# Functional Analysis of a Putative Dothistromin Toxin MFS Transporter Gene

**DOI:** 10.3390/toxins1020173

**Published:** 2009-12-08

**Authors:** Rosie E. Bradshaw, Zhilun Feng, Arne Schwelm, Yongzhi Yang, Shuguang Zhang

**Affiliations:** 1 Bio-Protection Research Centre, Institute of Molecular BioSciences, Massey University, Palmerston North, New Zealand; Email: fengzhilun@hotmail.com (Z.F.); schwelmo@gmx.de (A.S.); s.g.zhang@massey.ac.nz (S.Z.); 2 Biotechnology Research Institute, Qinghai Academy of Agriculture and Forestry, Xining, China; Email: zhmyyz03@yahoo.com.cn (Y.Y.)

**Keywords:** aflatoxin biosynthesis, *Dothistroma septosporum*, major facilitator superfamily, toxin transporter, red-band needle blight

## Abstract

Dothistromin is a non-host selective toxin produced by the pine needle pathogen *Dothistroma septosporum*. Dothistromin is not required for pathogenicity, but may have a role in competition and niche protection. To determine how *D. septosporum* tolerates its own toxin, a putative dothistromin transporter, DotC, was investigated. Studies with mutants lacking a functional *dotC* gene, overproducing DotC, or with a DotC-GFP fusion gene, did not provide conclusive evidence of a role in dothistromin efflux. The mutants revealed a major effect of DotC on dothistromin biosynthesis but were resistant to exogenous dothistromin. Intracellular localization studies suggest that compartmentalization may be important for dothistromin tolerance.

## 1. Introduction

Dothistromin toxin is produced by fungal pine pathogens in the genus *Dothistroma* (class Dothideomycetes) and is responsible for the characteristic red banding seen in infected needles [1,2]. A taxonomic revision in 2004 split the pathogen *Dothistroma pini* into two new species: *D. septosporum*, that has a worldwide distribution, and *D. pini* that appears to be confined to a few regions in the Northern hemisphere [3]. In recent years the prevalence and severity of this disease has increased, with epidemics in Canada and Europe associated with climate change [4,5].

Dothistromin is a difuroanthraquinone toxin with a polyketide backbone. Its structure is similar to that of versicolorin B, a precursor of aflatoxin and sterigmatocystin [6]. Dothistromin is toxic not only to pine tissue, but also to a wide range of other eukaryotes and bacteria [7,8], hence is considered a non-host-selective toxin. The precise mode of action is not known but dothistromin can be reductively activated leading to the formation of the reactive oxygen species superoxide (O_2_^-^) and H_2_O_2_ [9]. 

Dothistromin was once suspected to have a major role in needle blight disease. However dothistromin-deficient mutants of *D. septosporum* made by disruption of the dothistromin polyketide synthase gene (*pksA*) were pathogenic to *Pinus radiata*, showing that dothistromin is not required for disease development. Instead the toxin may have a role in competition against other microorganisms in the needle environment, a hypothesis that is supported by competition studies *in vitro* [10]. But given that dothistromin is highly toxic to a wide range of fungal species, the question arises of how *D. septosporum* is able to tolerate its own toxin.

Many fungal toxin gene clusters include toxin transporter genes and some of these have been shown to have roles in virulence and/or self-protection against the toxins [11,12]. Transporters of the non-host-selective toxin cercosporin have been implicated in virulence, including the CFP transporter in *Cercospora kikuchii* [13] and CTB4 in *Cercospora nicotianae*  [14]. Disruption of genes encoding these transporters significantly lowered the amount of cercosporin produced in each case, suggesting a role in regulation of cercosporin biosynthesis. However, whilst the CFP mutants were sensitive to exogenously supplied cercosporin the CTB4 mutants showed normal levels of resistance [13,14,15]. Disruption of some transporter genes appears to have no effect on either production or efflux of the associated toxin, such as the AflT transporter encoded in the aflatoxin gene cluster [16]. It is likely that there is redundancy with other transporters able to complement the disruption mutations [12,16,17].

Genes associated with dothistromin biosynthesis were identified on the basis of their similarity to aflatoxin genes [18,19]. The *dotA* gene was the first to be identified, and encodes a ketoreductase with 80% amino acid identity to the aflatoxin Ver-1 (AflM) in *Aspergillus parasiticus.* Other genes found alongside *dotA* include *dotC* that encodes a putative dothistromin transporter in the major facilitator superfamily (MFS), with 14 transmembrane domains. DotC is similar in sequence to MFS proteins in other species in the Dothideomycetes. It has 53% amino acid identity with both the Fnx1 multidrug resistance protein of *Phytophthora tritici-repens* (XP_001938334) and a hypothetical protein (SNOG_04383) in *Phaeosphaeria nodorum* (XP_001794802). In contrast there is less than 32% amino acid identity to *Aspergillus parasiticus* AflT (AAS66020.1), *Cercospora kikuchii* CFP (AAC78076) and *Cercospora nicotianae* CTB4 (ABK64181.1).

The aim of this investigation was to analyse the function of DotC to determine if it has a role in tolerance, efflux and/or biosynthesis of dothistromin. Mutants with a targeted disruption of *dotC*, or a complemented strain with multiple copies of *dotC*, were studied along with strains expressing DotC-GFP or DotA-GFP fusion proteins in order to determine the location and role of DotC in *D. septosporum*.

## 2. Results and Discussion

### 2.1. Production and characterization of *D. septosporum dotC* mutants and complemented strains

**Figure 1:**
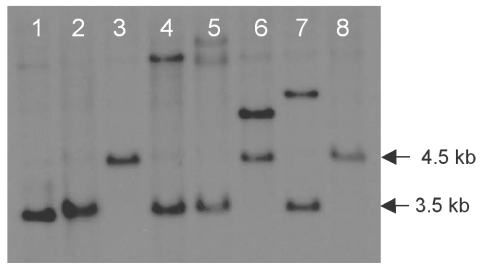
KpnI-digested genomic DNA of *D. septosporum* wild-type (lanes 1 & 2) or transformed with pR260 (lanes 3-8) and hybridized with a *dotC* coding region probe. KpnI sites flank the *dotC* gene, hence replacement of a 1.4 kb central portion of *dotC* with a 2.4 kb hygromycin resistance gene cassette increased the *dotC*-containing KpnI fragment size from 3.5 kb (wild-type) to 4.5 kb (*dotC* replacement). The *dotC* replacement mutants FJT16 and FJT15 are shown in lanes 3 and 8 respectively. Lane 6 contains another *dotC* mutant that had an additional ectopic integration of the pR260 plasmid.

*D. septosporum* strains transformed with a *dotC* replacement construct (pR260) were selected by hygromycin resistance. Two out of 26 hygromcyin resistant colonies (named FJT15 and FJT16) showed correct targeted replacement of the *dotC* gene when tested by PCR (results not shown) and Southern blotting (Figure 1). Other transformants had ectopic integration of the construct or vector integration only in the 3' region of *dotC*. The Southern blot also confirmed that there is only one copy of *dotC* in the *D. septosporum* genome. The *dotC* mutant FJT15 was subsequently transformed with a complementation construct (pR282) to restore *dotC* function and integration of the construct in the complemented strain FJT93 was confirmed by PCR (results not shown). The copy number of *dotC* in the complemented strain was estimated by real-time PCR to be 10 copies, making FJT93 a DotC-overproducing strain.

No significant difference was seen between sporulation of the *dotC* mutants FJT15 (1.48 ± 0.32 ± 10^6^ spores/mL; P = 0.725) and FJT16 (1.17 ± 0.29 ± 10^6^ spores/mL; P = 0.706) compared to the wild type NZE10 (1.32 ± 0.29 ± 10^6^ spores/mL). Likewise the radial growth rate of the *dotC* mutants was similar to that of the wild type with FJT15 (0.60 ± 0.03 mm/day; P = 0.092) and FJT16 (0.58 ± 0.02 mm/day; P = 0.182) not significantly different from the wild type (0.54 ± 0.02 mm/day). The *dotC*-complemented strain FJT93 grew slightly slower than the wild-type but in these experiments the difference was not significant (0.50 ± 0.02 mm/day; P = 0.119).

### 2.2. Resistance of mutants to exogenous dothistromin

To determine if DotC confers a protective effect against exogenously supplied toxin, the ability of *dotC* mutants to grow in the presence of dothistromin was investigated (Table 1). Although there was some variability in growth rates, as often seen with *D. septosporum*, none of the strains appeared to be strongly inhibited by even the highest concentration of dothistromin. Colonies on 100 μM dothistromin grew to >96% of the diameter of colonies grown without dothistromin. This concentration of dothistromin is in excess of levels usually secreted by the strains (5-11 μM for NZE10 and 20-40 μM secreted by FJT93). Hence it appears that DotC is not required for resistance to dothistromin in *D. septosporum*. Similarly in *C. nicotianae* CTB4 mutants showed normal levels of resistance to exogenously supplied cercosporin, although disruption of other putative toxin pump genes in *Cercospora* spp. (*CFP* in *C. kikuchii* and *ATR1* and *CnCFP* in *C. nicotianae*) had some effect on cercosporin resistance [13,14,15,16,17].

In these experiments using a total diameter calculation the complemented (DotC-overproducing) FJT93 strain did show significantly slower growth than the wild type (P < 0.01) in all concentrations of dothistromin, in contrast to the earlier study. 

**Table 1:** Growth of *D. septosporum* in the presence of exogenous dothistromin (doth). Mean radial growth (mm) ± standard error shown for two independent experiments each with 10 replicates. *Significant differences between growth of each strain with dothistromin compared to without dothistromin (P < 0.05) are indicated by asterisks.

Strain	0 μM doth	20 μM doth	40 μM doth	100 μM doth
NZE10 (wildtype)	14.7 ± 0.19	14.6 ± 0.27	14.0 ± 0.11*	14.2 ± 0.12*
FJT15 (Δ*dotC*)	15.5 ± 0.43	16.1 ± 0.30	16.1 ± 0.36	16.7 ± 0.33*
FJT16 (Δ*dotC*)	17.3 ± 0.29	16.6 ± 0.23	16.4 ± 0.25*	16.9 ± 0.20
FJT93 (Δ*dotC* +*dotC*)	13.4 ± 0.21	12.6 ± 0.14*	13.2 ± 0.18	13.3 ± 0.21

### 2.3. Dothistromin production by *dotC* disruption mutants

Amounts of dothistromin secreted into broth, and remaining in the mycelium, were determined by ELISA following a standardised extraction procedure (Table 2). Strikingly, the *dotC* disruption mutants (FJT15 and FJT16) secreted only 4-7% of the amount of dothistromin secreted by the wild type NZE10 strain. The amounts of dothistromin in the mycelia were also lower in these mutants than the wild-type, but these differences were not significant, possibly due to large variation between replicates. 

It is possible that the lower levels of secreted dothistromin in the *dotC* mutants were a direct consequence of overall lower levels of dothistromin production. The amounts of dothistromin produced by the mutants (mycelium and broth combined) were only 8.5% and 4.4% of wild type levels for the FJT15 and FJT16 mutants respectively. However the right-hand column in Table 2 suggests a small, but not significant, difference in the percentage of dothistromin secreted: in the wild type over 90% of all dothistromin was secreted whilst in the *dotC* knockout mutants only 80-83% was secreted. Thus there is only weak evidence for a role in dothistromin transport for DotC.

**Table 2:** Dothistromin as measured by ELISA from broth (secreted dothistromin) and mycelium (not secreted) extracted from the same flasks using the same extraction method (n = 3). For mycelium the amounts shown are dothistromin per mg dry weight. For broth the amounts of dothistromin have been calculated to show how much dothistromin was secreted into the broth by each mg of mycelium in the flask. *Significant differences from NZE10 values (P < 0.05) are indicated by an asterisk.

Strain	Dothistromin in broth		Dothistromin from mycelia		% Doth secreted broth/(myc + broth)
Dothistromin Mean ± SE (ng/mg DW)	% of WT (NZE10)	Dothistromin Mean ± SE (ng/mg DW)	% of WT (NZE10)
NZE10 (wildtype)	319.8 ± 30.3	100	30.5 ± 15.3	100	91.3%
NZE7 (wildtype)	249.1 ± 32.8	77.9	26.8 ± 16.6	88.1	90.3%
FJT15 (Δ*dotC*)	23.8 ± 5.0*	7.4	6.1 ± 2.2	20.1	79.5%
FJT16 (Δ*dotC*)	12.9 ± 2.7*	4.0	2.6 ± 0.6	8.4	83.4%
FJT93 (Δ*dotC* + *dotC*)	1716.6 ± 507.9	536.8	1105.3 ± 348.2*	3629.9	60.8%*

The lower levels of dothistromin in the *dotC* mutants strongly suggest that DotC is required for wild-type levels of dothistromin biosynthesis. DotC is a close match to MFS proteins, with no evidence for a DNA binding domain. A dramatic reduction in toxin production was also seen in CFP mutants of *Cercospora kikuchii*, with less than 5% of wild type levels of cercosporin produced [13], and also to CTB4 and ATR1 mutants of *C. nicotianae* with less than 35% and 25% of wild type cercosporin respectively [14,17]. 

The observation that some dothistromin was still secreted in the *dotC* mutants suggests that other transporters are involved. Multiple transporters with overlapping roles have been reported in several fungi. For example in *C. nicotianae* the ABC transporter ATR1 was shown to have a major role in cercosporin production and, along with another MFS transporter CnCFP, contributed to auto-resistance to cercosporin [17]. 

The *dotC* complemented mutant (FJT93) produced and secreted very high levels of dothistromin. The secreted levels were five-fold higher than those seen with the wild-type, but the variability between replicates was high. The amount of dothistromin secreted from FJT93 was significantly higher than the combined results from wild type strains NZE7 and NZE10 (P = 0.0037) but not compared to NZE10 alone (P = 0.0516). The levels of non-secreted dothistromin remaining in the mycelium were extremely high in FJT93: more than 30-fold higher than the wild type. It appears there was a dramatic increase in dothistromin biosynthesis in FJT93 and that the cells were unable to secrete the dothistromin as quickly as it was being made: almost 40% of the total amount of dothistromin remained in the mycelium. Since FJT93 only showed a slightly slower growth rate compared to the wild type (significant in only one of two trials) the high intracellular levels of dothistromin did not appear to be toxic. These high levels of toxin further suggest that dothistromin accumulation in the cell is not a primary feedback regulator of dothistromin biosynthesis. 

### 2.4. Dothistromin gene expression in *dotC* disruption and complemented mutants

**Figure 2:**
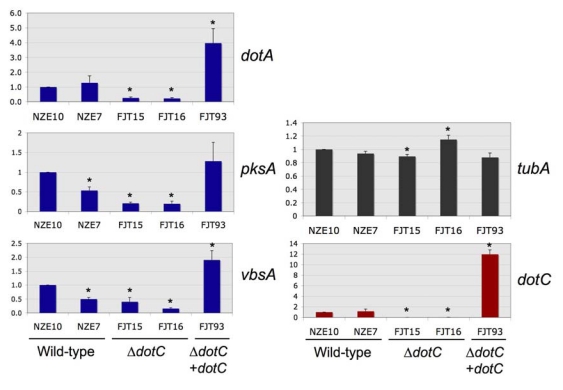
Expression of *dotC*, dothistromin genes *dotA*, *pksA* and *vbsA*, and beta tubulin (*tubA*, constitutive control) in wild type, *dotC* knockout (*∆dotC*) and complemented (*∆dotC* + *dotC)* strains. 18S ribosomal RNA was used as a reference gene for standardisation. Values are normalised expression ratios relative to the NZE10 wild type, shown as mean ± standard error (n = 6). *Significant differences from NZE10 (P < 0.05) are shown by an asterisk.

Real-time PCR was used to quantify *dotC* expression in the *dotC* mutants and complemented strain. As expected, the *dotC* disruption mutants FJT15 & 16 showed no *dotC* expression above background, as they lacked this single-copy gene. Expression of the *dotA* dothistromin biosynthetic gene was significantly reduced in the *dotC* mutants, to around 18-26% of levels in wild type strains (Figure 2 and Supplementary Table S3). It therefore seems likely that the lower levels of dothistromin made by the mutants are at least partly due to lower levels of biosynthetic enzymes. Both FJT15 and FJT16 also showed different *tubA* (constitutive control) expression levels compared to the wild type, but one had a higher and one a lower level of *tubA* so no consistent effects were noted. The *dotC* mutants showed lower levels of expression of the dothistromin biosynthetic genes *pksA* and *vbsA*, but the NZE7 wild type strain also had reduced levels of these transcripts compared to the NZE10 wild type. NZE7 and NZE10, like all New Zealand isolates of *D. septosporum*, are considered clonal but strain degeneration has sometimes been noted over time in axenic culture. The NZE7 strain was isolated from infected pine needles three years before NZE10 and the differences in gene expression may reflect this. Inter-strain differences were also noted between the independent *dotC* mutants FJT15 and FJT16, making the *pksA* and *vbsA* results less reliable than those for *dotA*.

In *C. nicotianae*, although disruption of the *CTB4* MFS gene led to significantly lower levels of cercosporin biosynthesis compared to the wild type, expression of the four cercosporin biosynthetic genes tested was not noticeably reduced in northern blots [14]. Likewise disruption of the *aflT* gene in *A. parasiticus* had no effect on the expression of aflatoxin biosynthetic genes, as well as no effect on aflatoxin production [16]. 

In contrast to the *dotC* mutants, the complemented FJT93 strain that contained multiple copies of *dotC* produced 12-fold more *dotC* transcript than the wild type, suggesting over-expression of DotC protein. It is possible that the *dotC* complementation construct may have integrated into a site that affects expression of dothistromin genes. However the increased gene expression is consistent with a role for DotC in regulating dothistromin biosynthesis and with the decline in dothistromin production seen in the *dotC* disruption mutants. Further to this, high intracellular concentrations of dothistromin in a strain that appears to make an excess of DotC adds support to the hypothesis that DotC is not the major efflux pump for dothistromin and suggests that there are other factors besides DotC limiting secretion.

The FJT93 complemented strain also over-expressed two of the dothistromin biosynthetic genes (*dotA* and *vbsA*), although levels were only 2-4 fold higher than the wild type. The fact that *pksA* gene expression was not significantly increased in the DotC and dothistromin over-expressing mutant is interesting because *pksA* encodes a polyketide synthase required for an early step of dothistromin biosynthesis. Since the *pksA* gene is essential for dothistromin production [20] this result suggests that PksA is not a major rate-limiting enzyme in dothistromin biosynthesis. 

### 2.5. Cellular localization of DotC-GFP and DotA-GFP fusion proteins

**Figure 3:**
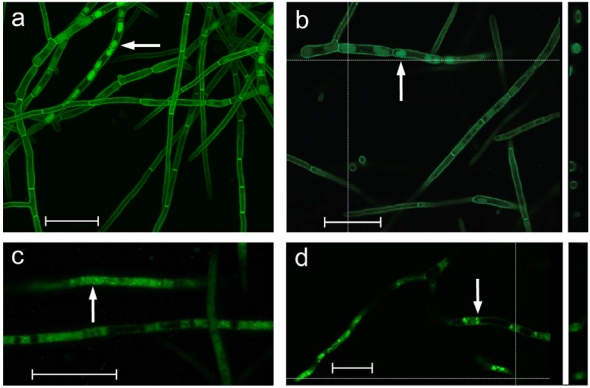
GFP fluorescence of *D. septosporum* transformed with *dotC-gfp* (**a**, **b**) or *dotA-gfp* (**c**, **d**). Confocal Laser Scanning Microscopy (CLSM) images on the left (a and c) are combined 0.2 μm Z-stacks. Images on the right (b and d) are single-layer snapshots, with vertical cross-section profiles illustrated on the right. White arrows indicate intracellular vesicles/vacuoles showing GFP-fluorescence. Vesicles in (c) are very small. Scale bar = 20 μm.

Confocal microscopy showed that the DotC-GFP fusion protein was located around the periphery of the cells (Figure 3), as would be expected for an MFS transporter. This is consistent with a role in secretion of dothistromin. However the images also revealed DotC-GFP localization in some intracellular regions. Cross-sections through these suggested that DotC-GFP was not confined to the perimeter but seen throughout. These GFP-containing regions varied in intensity and size but were reminiscent of vacuoles containing the aflatoxin pathway enzymes Ver-1 or Nor-1 fused to GFP [21,22]. Under aflatoxin-inducing conditions in *A. parasiticus* a large number of vesicles accumulated that were heterogeneous in both density and size, and a functional link of vesicles (defined as vacuolar-type structures with diameters < 2.5 μm) and vacuoles with aflatoxin biosynthesis was proposed [23]. 

Cellular localisation of the DotA dothistromin biosynthetic enzyme was also studied in a *D. septosporum* strain expressing a DotA-GFP fusion. The DotA-GFP fusion protein was predominantly cytoplasmic or located in small (Figure 3c) or larger (Figure 3d) vesicles, and showed similarities to the localization of GFP fusion genes of *A. parasiticus* aflatoxin genes, including the DotA homolog, Ver-1 [21,22]. The Ver-1 fusion protein was predominantly cytoplasmic at early stages of growth under aflatoxin-inducing conditions and found in the lumen of up to 80% of vesicle-like structures and vacuoles at later stages [21]. Studies with a Nor-1 fusion showed a similar trend [22]. In the present study *D. septosporum* cultures were observed after 4-5 days of growth (early exponential phase), so it is possible that DotA-GFP was in the early stages of moving to vacuoles. 

Although most of the DotC-GFP protein was localized in the cell membrane and therefore could be involved in efflux from the cell, its localisation in some intracellular organelles might suggest an additional role. We propose the hypothesis that mid to late stages of dothistromin biosynthesis occur in vacuoles as proposed for aflatoxin biosynthesis [21] and that this enables *D. septosporum* to tolerate high intracellular toxin levels as seen in the over-expressing strain FJT93. We further propose the hypothesis that one function of DotC may be to transport early-stage dothistromin biosynthetic intermediates from the cytoplasm into vacuoles, thereby affecting the rate of dothistromin production. This could explain the lower levels of dothistromin produced by *dotC* mutants and higher levels in the DotC over-producing strain. The CnCFP transporter in *C. nicotianae* was also speculated to have a role in transport between organelles and/or transport of early pathway components within the cell [17], although in this case no cellular localization studies were reported. 

There are some anomalies in our data that make interpretation difficult. Cross-sections of vacuoles showed DotC-GFP throughout some of the vacuoles, rather than just at the periphery as would be expected if DotC has a role in intracellular transport. Furthermore the *dotA-egfp* gene did not function as expected: it was transformed into a *dotA*^-^ dothistromin-deficient mutant strain (FJT2) and did not re-establish dothistromin production by complementation (results not shown). Therefore the observations of DotA-GFP expression might be artifacts, although the similarity to the aflatoxin biosynthesis proteins in *A. parasiticus* is intriguing. Further detailed studies of intracellular localization are required to determine the locations of dothistromin and the dothistromin biosynthetic enzymes, as well as the role of DotC in dothistromin biosynthesis. 

According to Amnuaykanjanasin [17], fungal transporters can be classified into three groups based on whether they have a major role in toxin efflux (group 3), self-protection (group 2) or both (group 1). In *C. nicotianae* the cercosporin transporters ATR1 and CnCFP were classified as group 1 whilst CTB4, the transporter located in the cercosporin gene cluster, was group 3. On the basis of our results the *D. septosporum* DotC that is encoded within the dothistromin gene cluster appears to be closest to a group 3 transporter as it may be involved in dothistromin efflux but has little or no effect on protection against exogenous dothistromin. If dothistromin is compartmentalized within the cell as hypothesized above, this may contribute to self-protection, providing one possible explanation for how the DotC-overproducing strain tolerated extremely high intracellular levels of dothistromin without any severe effects. 

An additional role that has now been noted with several fungal transporters is that of regulating toxin biosynthesis [17]. Our results clearly showed a dramatic decrease in dothistromin biosynthesis in DotC-deficient mutants and increase in a DotC-overexpressing strain. The most obvious way in which transporters might affect biosynthesis would be by a feedback mechanism due to intracellular toxin levels which would increase in mutants with deficient efflux systems. However our results suggest that a more complex type of regulation may occur. In the DotC-overproducing strain extremely high levels of dothistromin were accompanied by increased expression of dothistromin biosynthetic genes, rather than decreased expression as would be predicted from a simple dothistromin feedback regulation model. Compartmentalisation of toxin biosynthesis within the cell, as shown for *A. parasiticus* [21,22,23], and supported by our studies with *D. septosporum*, may prove to be an important factor in regulation of toxin biosynthesis. 

## 3. Materials and Methods

### 3.1. Strains and culture conditions

The wild-type *D. septosporum* strains NZE7 and NZE10(clonal forest isolates from New Zealand), and genetically modified strains derived from these, were routinely grown as previously described [10]. For radial growth measurements, 5-mm diameter agar plugs from 7-day old colonies were inoculated onto plates of Dothistroma Medium [24] and incubated at 22 °C. In three independent experiments, each with 3-7 replicates, radial growth was measured at intervals and a growth rate (mm/day) calculated over the 2-4 week period since inoculation in which growth rate was constant. To assess sporulation, colonies were grown as above but on Dothistroma sporulation medium [24]. After 3 weeks' incubation, two 5-mm diameter agar plugs of mycelium were taken from opposite edges of each colony (from three independent experiments, each with 3-7 replicates), vortexed in 200 μL 1% Tween 20, and spore concentrations determined using a cytometer. Results are presented as overall means ± SEM unless otherwise indicated. A two-tailed T-test was used to determine significant differences based on the null hypothesis of no difference between each mutant and the wild type.

### 3.2. Disruption and complementation of *dotC*

To disrupt the *dotC* gene, a replacement vector pR260 was prepared using a Multisite Gateway^TM^ system (Invitrogen) using methods previously described for *D. septosporumpksA* and *vbsA* genes [19,20]. Three entry clones were prepared by PCR amplification: (a) a 5' region of *dotC*, including nucleotides 19180 to 20313, numbered according to GenBank entry AF448056, (b) a hygromycin resistance (*hph*) selectable marker gene cassette and (c) a 3' region of *dotC*, including nucleotides 16783 to 17782. All primers used in this work are listed in Supplementary Table S1. The flanking regions were designed so that approximately 1.4 kb of the 1.9 kb *dotC* ORF would be replaced by the *hph* gene following recombination of the entry clones. The arrangement of the three fragments in the gene replacement plasmid, pR260, was confirmed by PCR and sequencing. Protoplasts of wild type *D. septosporum* NZE10 were transformed with plasmid pR260 using methods described previously [25]. 

Hygromycin-resistant transformants were single-spore purified and characterized using methods described previously [20]. DNA extracted from transformants was PCR-amplified to determine if *dotC* gene replacement had occurred. PCR primers were designed to amplify between the *hph* region of the pR260 construct and either 5' or 3' genomic regions flanking the *dotC* integration site (see Supplementary Figure S1 for details). Southern hybridisation of XbaI and BamHI-digested genomic DNA was used to confirm gene replacement using a digoxigenin (DIG)-labeled probe designed to cover the *dotC* ORF. 

Complementation of one of the *dotC* disruption mutants (FJT15) with the complete *dotC* gene was achieved using a 4.75 kb HindIII-NotI genomic DNA fragment that contained the complete *dotC* coding region and approximately 2 kb of upstream (intergenic) sequence. The fragment was ligated into the pBC-phleo vector that contains a phleomycin selectable marker [26] to form the plasmid construct pR282, then transformed into FJT15 using selection with 7 μg/mL phleomycin (Apollo Scientific Ltd., Stockport, UK). Transformants were single-spore purified and characterized by PCR. To determine the copy number of the *dotC* gene in the complemented strain FJT93, quantitative real-time PCR was carried out to compare *dotC* (target) and *tubA* (β-tubulin gene; normaliser) targets compared to the NZE10 wild type, using primers listed in Supplementary Table S2. Relative quantitative RT-PCR was performed using a LightCycler^®^ 480 SYBR Green 1 DNA Master kit (Roche). One μL of gDNA and 1 μL primer mix (50 μM each) were added to 8 μL Roche PCR mix and subjected to 45 cycles of PCR (10 s at 95 °C, 10 s at 57 °C, 20 s at 72 °C) with an acquisition temperature of 72 °C. Copy number was determined using the 2^ΔΔCT^ method and LightCycler^®^ 480 software with amplification efficiencies computed using standard curves.

### 3.3. Quantification of dothistromin and dothistromin resistance assay

Intracellular and secreted levels of dothistromin were determined for wild type, dotC mutants and complemented strains. Approximately 1×10^5^ conidia/mL, harvested from DSM plates, were inoculated into 25 mL of dothistroma broth (DB) (2.5% (w/v) Oxoid malt extract, 2% (w/v) Oxoid nutrient broth) in 125 mL conical flasks and incubated at 22 °C for 10 days with shaking at 150 rpm. Mycelium was harvested by vacuum filtration. For each strain, three biological replicates were assessed, and a known proportion of the mycelium from each flask was freeze-dried for dry weight determination. To extract intracellular dothistromin, 35-360 mg fresh weight of mycelium was disrupted using a Thermo Savant FastPrep FP120 cell disrupter (Thermo Electron Corporation, Milford, MA) with 300 μL H_2_O and ~20 μL beads for ten bursts of 20 s at speed level 5. An equal volume of chloroform was added, mixed for 4 hours at room temperature, the chloroform phase was collected then the aqueous phase re-extracted with chloroform four times more. The chloroform was evaporated and the residue re-dissolved in 200 μL ethyl acetate, then the solvent evaporated again and the residue that contained dothistromin finally dissolved in 20-50 μL DMSO. To maintain consistency, dothistromin was extracted from 5 mL of culture filtrate using the same method as described for the mycelium, except without the cell disruption step. Dothistromin concentrations per mL broth were determined using competitive ELISA [27] as previously described [24]. A direct comparison of intracellular and secreted dothistromin was made by calculating the amount of dothistromin secreted from 1 mg dry weight mycelium, based on the total amount of mycelium in a known volume in each flask. Dothistromin resistance was assessed by radial growth on Dothistroma Medium [24] containing either 0, 20, 40 or 100 μM dothistromin in DMSO, plus additional DMSO to make a final concentration of 0.08% DMSO in all plates. Five point-inoculations were made on each of two replicate plates using a well-sporulating inoculum, and toothpicks instead of the usual agar plug method, a modification suggested by Amnuaykanjanasin *et al* [17]. Radial growth measurements were made after 28 days growth at 22 °C. The experiment was done twice.

### 3.4. Real-time PCR analysis of dothistromin gene expression

RNA was extracted from samples taken from the same cultures used for dothistromin assays, using Trizol Reagent (Invitrogen) and cDNA synthesized by random hexamer primed reverse transcription using Superscript^TM^ III reverse transcriptase (Invitrogen). Relative quantitative RT-PCR was performed using a LightCycler^®^ 480 SYBR Green 1 DNA Master kit (Roche) as previously described [28]; (primers in Supplementary Table S2) but with amplification conditions modified to suit the LightCycler^®^ 480. Two μL of 10-fold diluted cDNA were added to 8 μL PCR mix and subjected to 45 cycles of PCR (10 s at 95 °C, then 30 s at each of 60 °C and 72 °C) with an acquisition temperature of 72 °C. Three technical replicates of each of two biological replicates were used for calibrator-normalized relative quantification analysis. The calibrator was 18S ribosomal RNA and the values were normalized by comparing to expression levels in the NZE10 wild type, with the constitutively-expressed *tubA* (beta-tubulin) gene as an internal control. 

### 3.5. Construction and analysis of DotC-EGFP and DotA-EGFP fusion strains

The *dotC-egfp* and *dotA-egfp* fusion genes were obtained using three-step combinatorial PCR, using Platinum^®^Taq DNA Polymerase High Fidelity (Invitrogen). In the first step for the construction of the *dotC-egfp* fusion gene, a 3 kb DNA fragment (including the *dotC* ORF and a 1.1 kb upstream region) was PCR amplified from *D. septosporum* genomic DNA using primers dotCzf1 and dotCzf2 (Supplementary table S1). In the second step, an 0.8 kb fragment containing the *egfp* (green fluorescent protein) gene was amplified from plasmid pPN82 [29] using primers dotCzf3 and pPN81-2978rev. Primers dotCzf2 and primer dotCzf3 used in steps 1 and 2 were complementary and designed to match both the end of *dotC* and beginning of *egfp* coding regions, but with the stop codon of *dotC* eliminated to allow translation read-through into *egfp*. The third PCR used products from the first two PCR reactions as template with dotCzf1 and pPN81-2978rev primers to amplify the complete fusion construct. The accuracy of the construct was verified by sequencing and it was cloned in a pBlueScript vector along with a hygromycin resistance (*hph*) selectable marker cassette (as above) to form plasmid pR265. Protoplasts of *D. septosporum* were transformed with plasmid pR265 as above. Purified transformants were screened for the presence of the *dotC-egfp* construct using PCR primers targeted to *dotC* (DSCE1) and *egfp* (egfp-rev) and by Southern hybridisation of XhoI-digested genomic DNA with a 420 bp DIG-labelled *egfp* gene fragment using methods described previously [20]. 

In the first step of the construction of the *dotA-egfp* fusion gene a 2kb DNA fragment (including the *dotA* ORF and a 1.1 kb upstream region) was PCR amplified from *D. septosporum* using primers PdotA-fus and egfp-dota-c. In the second step a 736 bp product was PCR amplified from plasmid pPN81 (a modified version of pPN82 [29] without the *trpC* terminator) using primers dota-egfp-c and TtrpC-fus. The primers dotA-egfp-c and egfp-dotA-c were complementary and designed to match both the end of the *dotA* and the beginning of the *egfp* coding regions, but replacing the *dotA* stop codon by the start codon of the *egfp* gene. The third step used the first two PCR products as a template and pdotA-fus and TtrpC-fus primers to PCR amplify the complete *dotA-egfp* fusion gene. The PCR product was cloned into pGEMTeasy (Promega) according to the manufacturer’s instructions. The accuracy was verified by sequencing. A HindIII fragment from pPN82 containing the *hph* gene was inserted into a single restriction site resulting in vector pR261. The insertion of the *hph* gene reduced the upstream region of the *dotA* gene from 1130 to 1079 bp. Protoplast-based transformation was used to co-transform a dothistromin deficient strain *dotA*^-^ strain (FJT2) with pR261 and pBC-Phleo containing a selectable marker conferring phleomycin resistance [26], as described previously [10].

A strain of *D. septosporum* (FJT22) [28] containing a constitutively-expressed e*gfp* gene was used as a control. For microscopic analysis, spores of *D. septosporum* transformants were inoculated into 15 mL DB broth in Petri dishes and incubated for 4-5 days at 22 °C. Fluorescence of hyphal samples was firstly assessed using an Olympus BX51 microscope with excitation and emission filters set at 460-490 nm and 510-550 nm respectively. Samples were then analysed with a Leica TCS SP5 confocal laser scanning microscope with excitation at 405 nm using a diode laser, and GFP fluorescence recorded between 506 and 589 nm. The images shown were taken using a 63× objective with 1.64 zoom, in 0.2 μm steps and set at 1024 × 1024 pixels.

## 4. Conclusions

Whether DotC has a role in dothistromin efflux from the cell remains inconclusive. Firstly although mutants deficient in DotC secreted less dothistromin, and a slightly lower % of total dothistromin, than wild types, these results could have been due to overall lower levels of dothistromin biosynthesis. Secondly although DotC-GFP fusions suggested that DotC is located on the cell membrane, DotC does not appear to be the primary efflux transporter as *dotC* mutants can still secrete dothistromin. Furthermore a DotC-overproducing strain appeared unable to pump out the very high levels of dothistromin it produced, and had high intracellular toxin levels, suggesting rate limitation by other efflux systems. 

In general, transcription of dothistromin genes and dothistromin production decreased in DotC-deficient mutants and increased in a DotC-overproducing strain, suggesting an important role for DotC in regulation of dothistromin biosynthesis. Cellular localization studies supported the hypothesis that dothistromin biosynthesis occurs in vacuoles or vesicles as previously suggested for aflatoxin biosynthesis. It is possible that compartmentalization of dothistromin provides *D. septosporum* with a mechanism of self-protection against its own toxin, and may be the key to understanding how efflux pumps can contribute to the regulation of toxin biosynthesis.

## Supplementary Files

Supplementary File 1: Supplementary Material.pdf (PDF, 148 KB)
