# Supplementary material for: Functional Analysis of a Putative Dothistromin Toxin MFS Transporter Gene
Source: Toxins (Basel). 2009 Dec 8;1(2):173–87. doi: 10.3390/toxins1020173 (PMC3202781; doi:10.3390/toxins1020173)
Supplement: Supplementary File 1: — Supplementary Material.pdf (PDF, 148 KB) [file toxins-01-00173-s001.pdf]

Supplementary Figure S1.
PCR screening of the *dotC* disrupted transformants

a. Schematic map of PCR screening of *dotC* gene replacement transformants

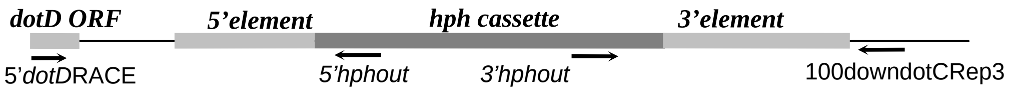

Primer positions for screening the transformants are indicated by arrows.

5' and 3' elements refer to the dotC gene

b. PCR screening results

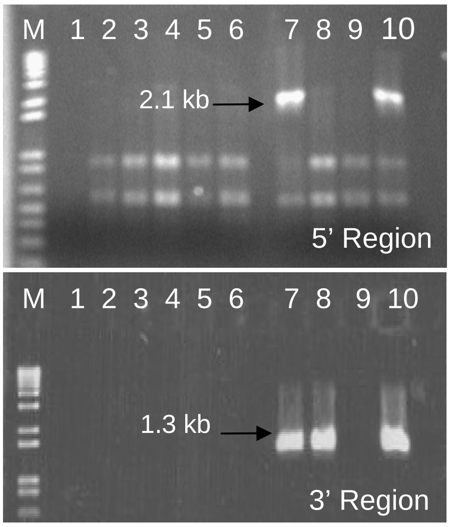

Top panel: PCR results for 5' region using primers *5'dotD* RACE and *5'hphout*. Transformants FJT15 and FJT16 gave 2.1 kb PCR products, indicating correct gene replacement at the 5' end of *dotC*.

Top panel: PCR results for 5' region using primers *3'hph* out and *100downdotCRep3*. Transformants FJT15 and FJT16, as well as FJT19 gave 1.3 kb PCR products, indicating correct gene replacement at the 3' end of *dotC*.

Lane M: 1 kb^{+} ladder
Lane 1: negative control (no DNA);
Lane 2: NZE10 wild type genomic DNA as template
Lanes 3-10: Genomic DNA template from transformants:
Lane 3: FJT 43
Lane 4: FJT 42
Lane 5: FJT 17
Lane 6: FJT 18
Lane 7: FJT 16
Lane 8: FJT 19
Lane 9: FJT 41
Lane 10: FJT15

Only FJT15 and FJT16 were confirmed as *dotC* gene replacement mutants.

Supplementary Table S1:
dotC knockout, complementation and *gfp*-fusion primers

Primer name	Lab. code	Sequence (5'to 3')	Purpose
dotC3'fwd	395	GGGGACAGCTTTCTTGTACAAAGTGG<br>GCACTCCAGACCAAGATCAAGCAGA	Prep dotC KO construct<br>pR260 Incl attB2
dotC3'rev	396	GGGGACACCTTTGTATAATAAAGTTG<br>GTCTGCATCGTGCGGTTGTACCTGT	Prep dotC KO construct<br>pR260 Incl attB3
dotC5'fwd	397	GGGGACAACTTTGTATAGAAAAGTTGATC<br>TTACGATGCGACTCGATGTGTG	Prep dotC KO construct<br>pR260 Incl attB4
dotC5'rev	398	GGGGACTGCTTTTTTGTACAAACTTG<br>CGGATCCTTCTCGGACAAGTTGTCG	Prep dotC KO construct<br>pR260 Incl attB1
100downdot<br>C-Rep3	385	TTGTGGCGAATCAGGATCCA	Screen dotC KO
3'hphout	36	TCCTTGAACTCTCAAGCCTACAG	Screen dotC KO &<br>complementation
5'hphout	35	GAATCTCCGGTGTGGAAGA	Screen dotC KO
5'dotD<br>RACE	608	TAGACGGCGAGGTCGCGAGAGAT	Screen dotC KO
dotCzf1	400	ATCTTACGATGCGACTCGATGTGT	Prep. dotC-gfp fusion
dotCzf2	401	TCCTCGCCCTTGCTCACCATGGACTTTTG<br>GGCCTTCTCCA	Prep. dotC-gfp fusion
dotCzf3	402	TGGAGAAGGCCCAAAAGTCCATGGTGAG<br>CAAGGGCGAGGA	Prep. dotC-gfp fusion
pPN81<br>2978rev	425	TCTCAACTCCGGAGCTGA	Prep. dotC-gfp fusion
DCSE1	386	GAGAGACCTTGCAAGATC	Screen dotC-gfp fusion and<br>complementation
egfp rev	693	AGAAGATGGTGCGCTCCT	Screen dotC-gfp fusion
PdotA-fus	219	GAACAGCCCGGGAGATTTGG	Prep. dotA-gfp fusion
egfp-dota-c	218	CTCGCCCTTGCTCACCATTCGGAAAGCA<br>CCACCGTC	Prep. dotA-gfp fusion
dota-egfp-c	217	GACGGTGGTGCTTTCCGAATGGTGAGCA<br>AGGGCGAG	Prep. dotA-gfp fusion
TtrpC-fus	220	ATACCCGGGTTACTTGTACAGC	Prep. dotA-gfp fusion
pUC/M13<br>fwd	29	CGCCAGGGTTTTCCCAGTCACGAC	Prep. dotC complementation
MF4151P3	99	GGACCAGAGGAACATACTTGG	Screen dotC<br>complementation
MF4152P2	103	CTATCATTGTCGCTTCGTAACG	Screen dotC<br>complementation
MF4152P4	105	AGACCAGCAGGCAGATGACAG	Screen dotC<br>complementation
MF4151P1	97	ACTTTCAGATGTCCATGGCAGC	Screen dotC<br>complementation

Supplementary Table S2: Real-time PCR primers

Gene/ purpose	Primer name	Lab. code	Sequence (5' to 3')	Amplicon Size cDNA(bp)	Amplicon Size gDNA(bp)
general ribosomal primer (qRT-PCR)	NS7	666	GAGGCAATAACAGGTCTGTGATGC	377	377
	NS8	667	TCCGCAGGTTCACCTACGGA		
vbsA (qRT-PCR)	rt VBS fw I	674	CCGAGCCACAAGAGGG	430	n/a*
	rt VBS rev I	675	CGGGTGAATGGGCTGA		
dotA (qRT-PCR)	rt dotA fw 1	691	CTGGTGATGAATTCGACCG	462	n/a
	rt dotA rev 1	692	AAGCACCACCGTCAATAC		
pksA (qRT-PCR)	rt pksA rev 1	695	CGAACAGAACTACCGACC	409	n/a
	rt pksA fw 1	696	CATTATGTCGTCCGAGCAC		
tubA (qRT-PCR)	rt TUB fw I	709	CCGGCGTGTACAATGG	392	n/a
	rt TUB rev I	710	CATGCGGTCTGGGAAC		
dotC (qRT-PCR)	rt DotC fw1	711	GCTTCTTCATCATCGGCG	518	518
	rt DotC rev I	712	TGGTCCGTTGCCGATAC		
tubA (gDNA copy no.)	rt TUB fw II	728	CGGTATGGGTACGCTCT	-	321
	TUB 4	227	TTGCGGAGATCACTGTTGAGCTG		
dotC (gDNA copy no.)	MF4151p3	99	GGACCAGAGGAACATACTTGG	-	438
	MF4152p4	105	AGACCAGCAGGCAGATGACAG		

*n/a One of the primers in each case was designed to flank intron sequence so no amplification could occur with genomic DNA template

Supplementary Table S3:
Expression of dothistromin genes measured by real-time PCR

	Wild type		*dotC* disruption		complemented
gene	NZE10	NZE7	FJT15	FJT16	FJT93
dotA	1	1.28 ± 0.47	0.26 ± 0.06	0.23 ± 0.05	3.96 ± 0.98
pksA	1	0.53 ± 0.09	0.21 ± 0.03	0.20 ± 0.07	1.28 ± 0.48
vbsA	1	0.50 ± 0.06	0.40 ± 0.16	0.16 ± 0.03	1.90 ± 0.34
tubA	1	0.94 ± 0.03	0.89 ± 0.03	1.15 ± 0.06	0.88 ± 0.07
dotC	1	1.15 ± 0.44	0.00 ± 0.00	0.01 ± 0.01	11.95 ± 0.84

Expression of *dotC*, dothistromin genes *dotA*, *pksA* and *vbsA*, and beta tubulin (*tubA*, constitutive control) in wild type, *dotC* disruption and complemented strains. These are the values corresponding to the chart in Figure 2. The 18S ribosomal RNA was used as a reference gene for standardization in real-time PCR. Values are normalised expression ratios relative to the NZE10 wild type (from which *dotC* disruptant strains were derived), shown as mean ± standard error (n = 6). Significant differences from NZE10 ($P<0.05$) are shown by grey shaded boxes. Significant differences from NZE7 ($P<0.05$) follow the same pattern except that FJT15 *dotA, vbsA* and *tubA* are not significantly different from NZE7 values.
